# Multidisciplinary management based on clinical nursing pathway model for the treatment of hypertensive intracerebral hemorrhage: A randomized controlled trial

**DOI:** 10.1097/MD.0000000000037644

**Published:** 2024-03-22

**Authors:** Lan Zhang, Tingting Shen, Yan Zhou, Xing Xie, Jing Wang, Haixiao Gao

**Affiliations:** a Department of Neurosurgery Ward 3, Xingtai Third Hospital, Xingtai Cardiovascular Disease Hospital, Xingtai City, Hebei Province, China.

**Keywords:** clinical nursing pathway, hypertensive intracerebral hemorrhage, multidisciplinary management

## Abstract

**Objective:**

To explore the effectiveness of multidisciplinary management based on a clinical nursing pathway model for the treatment of hypertensive intracerebral hemorrhage (HICH).

**Methods:**

A total of 124 patients with HICH admitted to our hospital between February 2021 and June 2023 were selected as research subjects in this randomized, controlled, unblinded study. They were divided into Control-group and Study-group using a random number table method, with 62 cases in each group. The Control-group received routine care and the Study-group adopted a multidisciplinary management approach based on the clinical nursing pathway model. A multidisciplinary intervention group including 1 attending physician, 1 psychotherapist, 1 nutritionist, 1 rehabilitation specialist, and 4 responsible nurses was constructed. From preoperative to postoperative day, patients were provided with psychological intervention, health education, respiratory tract management, and specific care for patients who were restless. One to 3 days after operation, the patients and their family members were guided in basic postoperative care and nutrition care. From the 4th day after surgery to the 1st day before discharge, patients were guided for rehabilitation exercises. Patients also received discharge advices upon discharge. Activities of daily living, neurological function, stress response indicators, incidence of complications, and nursing satisfaction before and after the intervention were compared between the 2 groups.

**Results:**

After the intervention, the activities of daily living and neurological function of the 2 groups were significantly improved compared to before the intervention, and the Study-group was significantly higher than the Control-group (*P* < .05). After intervention, the levels of stress response indicators in both groups significantly decreased compared to before the intervention, and the Study-group was significantly lower than the Control-group (*P* < .05). The incidence of complications in the Study-group (3.23%) was lower than that in the Control-group (15.00%) (*P* < .05). Nursing satisfaction in the Study-group (95.16%) was higher than that in the Control-group (83.33%) (*P* < .05).

**Conclusions:**

Our findings indicate that adopting a multidisciplinary management approach based on clinical nursing pathways to intervene in patients with HICH can reduce stress response levels, reduce the risk of complications, and facilitate the recovery of neurological function and activities of daily living with high patient satisfaction.

## 1. Introduction

Hypertensive intracerebral hemorrhage (HICH) is a common clinical disease.^[1]^ In recent years, the incidence rate of HICH has continued to increase, and its disability and mortality rates are high, posing a huge threat to the physical and mental health and quality of life of patients.^[1,2]^ Minimally invasive hematoma removal surgery plays an important role in the treatment of HICH by reducing intracranial pressure, disability, and mortality risks. Effective nursing interventions during treatment are of great significance in ensuring good disease outcomes.^[3,4]^

Routine nursing measures for HICH provide only basic interventions. The lack of systematic and targeted-specific measures and the insufficient subjective initiative of nursing staff make it difficult to achieve the desired results.^[5,6]^ The clinical nursing pathway is a proactive, systematic and planned schedule of nursing interventions for a specific group of patients, with time as the horizontal axis, and health guidance, clinical examination and treatment as the vertical axis, so as to maximize the satisfaction of patients’ care needs and provide high-quality nursing services to patients.^[7,8]^ In addition, the condition of HICH is complex, and rehabilitation treatment must involve multiple disciplines. Multidisciplinary management, on the other hand, is based on knowledge of multiple disciplines such as anesthesia, surgery, and nursing. Specific intervention measures are formulated based on the specific condition of patients, and corresponding nursing is conducted based on the clinical status of patients to reduce the occurrence of complications and shorten the rehabilitation process.^[9]^

Based on this, this study aimed to study patients with HICH admitted to our hospital, in order to clarify the application value of multidisciplinary management based on the clinical nursing pathway model in diseases, and provide a practical reference for nursing management of HICH.

## 2. Materials and methods

### 2.1. General information

A total of 124 patients with HICH admitted to our hospital between February 2021 and June 2023 were selected as research subjects. They were divided into control and study groups using a random number table method, with 62 cases in each group. During the treatment process, the Control-group received routine nursing care and the Study-group adopted a multidisciplinary management approach based on the clinical nursing pathway model. This was a randomized, controlled, unblinded study as a blinded control-group is not possible for this type of research and intervention.

### 2.2. Eligibility criteria

#### 2.2.1. Inclusion criteria.

- Meets the diagnostic criteria for HICH;^[10]^
- All patients underwent minimally invasive hematoma removal surgery.

#### 2.2.2. Exclusion criteria.

- Individuals with shock or intracranial infection;
- Cerebral hemorrhage caused by factors such as aneurysm and trauma;
- Individuals with coagulation disorders;
- Individuals with consciousness disorders or mental system disorders;
- Individuals with brain tumors;
- Women during lactation and pregnancy;
- Long-term postoperative coma.

### 2.3. Intervention measures

All care for patients in both groups was provided by the same nurses.

#### 2.3.1. Control-group.

The patients received routine care and strictly followed medical advice for symptomatic treatment such as hemostasis, blood pressure reduction, and low-flow oxygen inhalation. Moreover, the patients were introduced in detail to the pathogenesis, treatment measures, prognosis, possible complications, and daily precautions of HICH. The patients’ vital signs were closely monitored, and corresponding treatment measures were taken immediately in case of abnormalities. The drainage tubes after surgery were properly fixed, and the color and amount of drainage fluid were monitored.

#### 2.3.2. Study-group.

In addition to measures implemented in the Control-group, a multidisciplinary management approach based on the clinical nursing pathway model was adopted. An intervention group was constructed, including 1 attending physician, 1 psychotherapist, 1 nutritionist, 1 rehabilitation specialist, and 4 responsible nurses, to inform patients of the significance of research, knowledge of nursing care for HICH, clinical nursing pathways, theoretical knowledge of multidisciplinary management, and intervention techniques. Clinical interventions were implemented. From preoperative to postoperative day: Psychological intervention involved a psychotherapist introducing medical staff and ward environment to patients and their families, assisting them in undergoing imaging examinations, mastering patients’ emotional characteristics through facial expression observation, face-to-face communication, and other forms; inquiring about negative emotions and the causes of emotional fluctuations; and implementing targeted psychological intervention; Patients were provided with health education to learn more about the causes, pathogenesis, and treatment measures of the disease, and past successful cases that have achieved good surgical results were explained to assist patients in building rehabilitation confidence; Respiratory tract management was performed to clean secretions in the respiratory tract through manual sputum discharge, with suction devices to ensure smooth airway flow. During surgery, the patient chest movement and lung function were closely monitored and reported if any abnormalities were found during the anesthesia recovery period. Patients lied flat, with the head tilted back or to one side, and the jaw supported. Tracheal intubation, laryngeal mask, or oral ventilation tube were performed to maintain ventilation; For those who were restless, the tension band was fixed to ensure appropriate tightness and avoid causing discomfort to the patient; after the surgery, the patient bedside was raised by 30°, and when the patient regained consciousness, the throat was moistened with an appropriate amount of warm saline, and vibration or back tapping was used to promote sputum excretion. One to 3 days after the operation, the patient was assisted in changing the body position once every 2 hours during bed rest, and the patient was assisted in relieving pain through ice compress apparatus, acupuncture and moxibustion therapeutic apparatus, etc.; The patient family members were guided to regularly massage the compressed area for 30 minutes per session, and warm saline solution was used to wipe the body; After the patient first evacuation, a nutritionist guided them to consume a small amount of liquid food, and gradually transitioned to semi-liquid food and regular food based on tolerance and body recovery; And based on the patient personal preferences, a diet rich in dietary fiber and balanced nutrition was developed for them. From the 4th day after surgery to the 1st day before discharge, the rehabilitation specialist assisted the patient in placing good limb positions through on-site demonstrations, playing multimedia videos, displaying pictures, and engaging in passive movements of the limbs. Once the condition stabilizes, the patients were guided to roll over and balance sitting exercises, and gradually engage in single-legged birds, swallow balance, and pelvic lateral swing exercises to promote the recovery of core balance. Upon discharge, patients were advised to avoid dryness and anger, quit smoking and alcohol, measure blood pressure regularly every day, adhere to scientific exercise, and follow medical advice.

### 2.4. Outcome measures

The primary outcome of this study was neurological function at discharge. The secondary endpoints were activities of daily living, levels of stress response indicators, incidence of complications, and nursing satisfaction.

Calculating the neurological function and activities of daily living of both groups before and after intervention. The neurological function was evaluated using the National Institutes of Health Stroke Scale (NIHSS), including limb ataxia, consciousness, language, sensation, etc. The score range was 0 to 42 points, and the lower the score, the better. Activities of daily living were evaluated using the Barthel index (BI) scale, including urination, dressing, walking, and bathing. The score ranges from 0 to 100 points, with higher scores being better. The levels of stress response indicators (norepinephrine [NE], epinephrine [E], and cortisol [Cor]) before and after the intervention in both groups. Fasting venous blood was extracted and centrifuged (4000 rpm, 15 minutes), the supernatant was collected, NE and E levels were measured by liquid chromatography, and Cor levels were measured by radioimmunoassay. The incidence of complications such as malnutrition, infection, rebleeding, and constipation were calculated for both groups. Statistical analysis of nursing satisfaction between the 2 groups and self-assessment using a self-designed scale, including nursing attitude, nursing quality, etc, a total of 100 points, divided into very satisfied (90–100 points), satisfied (70–89 points), and dissatisfied (≤69 points); satisfied and very satisfied were included in the total satisfaction.

### 2.5. Statistical analysis

All data analyses were conducted using SPSS software (version 26.0; IBM Corp., Armonk, NY). Normality of the data was evaluated using the Shapiro–Wilk test. The data of normal distribution are represented by mean ± standard deviation, and the t-test was used, and the counting data were represented by the number of use cases using the chi-square test. *P* < .05 suggesting that the difference was statistically significant.

## 3. Results

**Figure 1.**
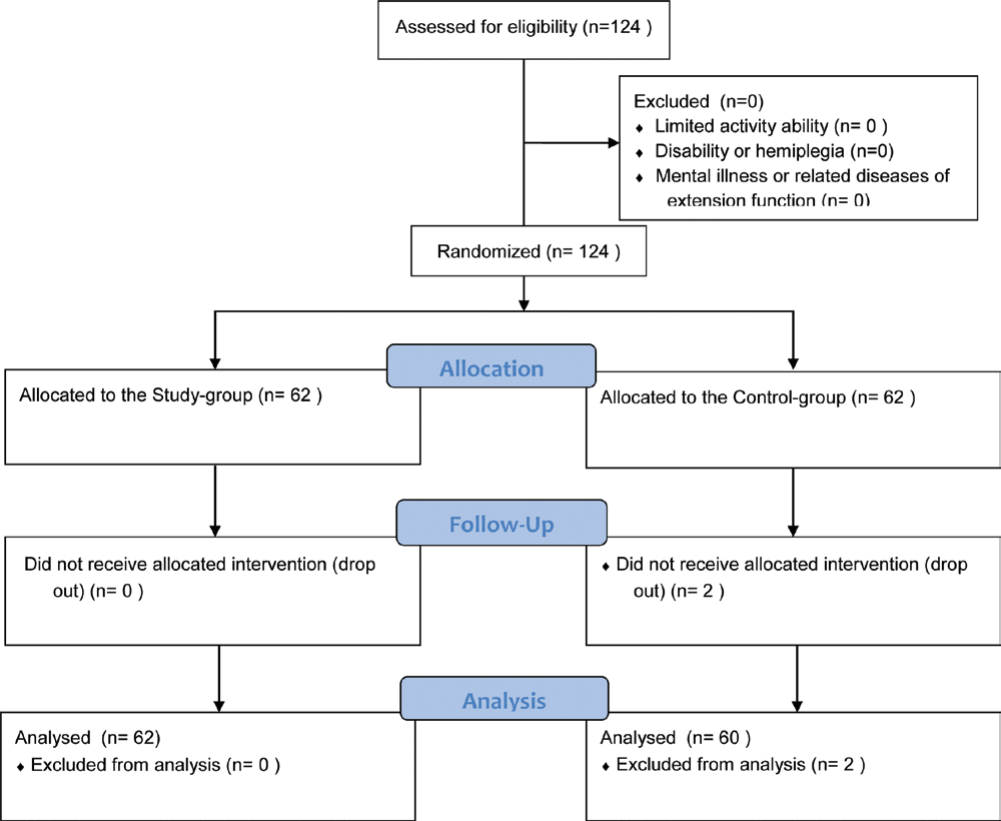
Guidelines flow diagram.

Baseline Characteristics: A total of 124 patients were recruited between February 2021 and June 2023. Two patients were excluded from this study (2 patients withdrew midway through the study); ultimately, 122 patients completed the study. Among them, there were 62 patients in the Study-group and 60 were in the Control-group (Fig. 1). There was no significant difference in baseline data between the 2 groups of patients (*P* > .05) (Table 1).

**Table 1:** Comparison of baseline data between 2 groups of patients [n(%)].

Group	Gender (male/female)	Age (yr)	BMI (kg/m²)	Bleeding site			
				Lobe of brain	Cerebellum	Thalamus	Basal ganglia area
Study-group (n = 62)	36/26	62.32 ± 7.63	24.20 ± 2.87	7 (11.29)	11 (17.74)	14 (22.58)	30 (48.39)
Control-group (n = 60)	28/32	61.33 ± 8.94	24.99 ± 2.47	13 (21.67)	9 (15.00)	13 (21.67)	25 (41.66)
* χ^2^/t*	1.588	0.658	−1.625	2.459			
* P*	.208	.512	.107	.483			

BMI = body mass index.

Comparisons of outcomes between the 2 groups: Before intervention, there was no significant difference in BI and NIHSS scores between the 2 groups (*P* > .05); After intervention, the BI levels in both groups were significantly higher than before intervention, and the Study-group was significantly higher than the Control-group; And NIHSS significantly decreased compared to before intervention, and the Study-group was significantly lower than the Control-group (*P* < .05) (Table 2). Before intervention, there was no significant difference in NE, E, and Cor levels between the 2 groups (*P* > .05); After intervention, the levels of NE, E, and Cor in the 2 groups were significantly reduced compared to before intervention, and the Study-group was significantly lower than the Control-group (*P* < .05) (Table 3). The incidence of complications in the Study-group (3.23%) was significantly lower than that in the Control-group (15.00%) (*P* < .05) (Table 4). Nursing satisfaction in the Study-group (95.16%) was significantly higher than that in the Control-group (83.33%) (*P* < .05) (Table 5).

**Table 2:** Comparison of 2 groups of BI and NIHSS (points).

Time	Group	n	BI	NIHSS
Before intervention	Study-group	62	44.85 ± 5.09	20.32 ± 3.60
	Control-group	60	45.33 ± 5.91	19.67 ± 3.79
	*t*		−0.480	0.981
	*P*		.632	.328
After intervention	Study-group	62	71.84 ± 9.16*	9.65 ± 3.05*
	Control-group	60	66.33 ± 9.43*	11.85 ± 2.74*
	*t*		3.271	−4.197
	*P*		.001	<.001

Compared with the same group before intervention,

* *P* < .05.

BI = Barthel index; NIHSS = National Institutes of Health Stroke Scale.

**Table 3:** Comparison of stress response index levels between the 2 group.

Time	Group	n	NE (nmol/L)	E (nmol/L)	Cor (ng/L)
Before intervention	Study-group	62	376.32 ± 30.64	233.95 ± 25.69	260.03 ± 25.12
	Control-group	60	381.60 ± 34.85	230.77 ± 29.16	257.00 ± 29.36
	*t*		−0.889	0.641	0.614
	*P*		.376	.523	.506
After intervention	Study-group	62	196.48 ± 18.81*	109.97 ± 16.14*	117.98 ± 16.16*
	Control-group	60	221.07 ± 16.70*	146.25 ± 19.16*	147.58 ± 20.23*
	*t*		−7.624	−11.326	−8.943
	*P*		<.001	<.001	<.001

Note: Compared with the same group before intervention,

* *P* < .05.

Cor = cortisol, E = epinephrine, NE = norepinephrine.

**Table 4:** Comparison of complications between the 2 groups [n(%)].

Group	n	Malnutrition	Infection	Rebleeding	Constipation	Total occurrence rate
Study-group	62	1 (1.61)	0 (0.00)	0 (0.00)	1 (1.61)	2 (3.23)
Control-group	60	3 (5.00)	2 (3.33)	1 (1.67)	3 (5.00)	9 (15.00)
*χ^2^*						5.153
*P*						.023

**Table 5:** Comparison of nursing satisfaction between the 2 groups [n(%)].

Group	n	Very satisfied	Satisfied	Dissatisfied	Total satisfaction rate
Study-group	62	38 (61.29)	21 (33.87)	3 (4.84)	59 (95.16)
Control-group	60	27 (45.00)	23 (38.33)	10 (16.67)	50 (83.33)
*χ^2^*					4.481
*P*					.034

## 4. Discussion

The results of this study show that a multidisciplinary management plan based on the clinical nursing pathway model can help promote neurological function and activities of daily living of patients with HICH, reduce the risk of complications, and ensure good disease outcomes. The research results of Zeng et al^[11]^ indicated that adopting a clinical nursing pathway model to intervene in patients with HICH in the basal ganglia region can effectively reduce the occurrence of complications and alleviate patients’ depression and anxiety. Zhou Yun^[12]^ intervened in HICH patients after minimally invasive hematoma removal surgery using a clinical nursing pathway and found that the NIHSS score of patients was lower than that of the Control-group who received routine care, while the BI score was higher than that of the Control-group; the incidence of complications was only 5.26%, lower than the Control-group (28.95%). Li et al^[13]^ applied multidisciplinary collaborative nursing processes to the emergency treatment of HICH, and the results showed that the patient emergency steps took a shorter time than the Control-group, and the NIHSS and Functional Independence Assessment (FIM) scores were better than the Control-group. These scholars have confirmed the clinical application value of clinical nursing pathways and multidisciplinary management models, which is consistent with the results of this study. Zhijuan et al^[14]^ investigated diabetes patients with malignant tumors by integrating clinical nursing paths and multidisciplinary cooperation programs, proving that it can deepen patients’ correct understanding of their own diseases and shorten the process of disease rehabilitation. The results of the above studies indicate that adopting a multidisciplinary management model based on clinical nursing pathways is feasible and effective for implementing interventions for patients with HICH.^[11–14]^ The main reason for this is that in multidisciplinary management based on the clinical nursing pathway model, health education and successful case explanation can deepen patients’ correct understanding of their own diseases and treatment plans, avoid anxiety, tension, fear, and other psychological factors caused by improper cognition, thereby alleviating stress reactions caused by negative emotions. Close monitoring of the patient vital signs during the perioperative period and cooperation with physicians to reduce intracranial pressure and other interventions can alleviate neurological damage caused by intracranial hypoxia and ischemia as soon as possible, and gradual functional rehabilitation exercises based on the specific state of the patient after surgery are beneficial for promoting the recovery of the patient activities of daily living.^[15,16]^ At the same time, in multidisciplinary management based on the clinical nursing pathway model, time is used as the horizontal axis, and intervention measures are used as the vertical axis to provide high-quality and orderly care services to patients through procedural and standardized intervention plans; The medical staff involved in disease rehabilitation treatment participate in disease management together, which can achieve collaborative purposes. Staff from various fields collaborate to develop intervention measures and provide patients with comprehensive psychological intervention, exercise guidance, dietary guidance, etc. Not only can it alleviate the psychological pressure of patients, but compared to conventional nursing measures, it is more scientific, humane, and professional, ensuring the quality of nursing services.^[17,18]^

The results of this study showed that the levels of stress response indicators in the Study-group were significantly lower than those in the Control-group, indicating that multidisciplinary management measures based on clinical nursing pathway models can help reduce the degree of stress response in patients with HICH. Conventional nursing measures lack sufficient attention to the patient psychological state, coupled with insufficient understanding of their own diseases and treatment measures, making them highly susceptible to severe negative emotions and exacerbating the degree of perioperative stress response; In a multidisciplinary management plan based on the clinical nursing pathway model, health education and psychological care can effectively regulate the patient physical and mental state, receive rehabilitation treatment in a good state, and reduce the degree of stress response.^[7,19]^ The results of this study also showed that nursing satisfaction of the Study-group was significantly higher than that of the Control-group, indicating that multidisciplinary management measures based on the clinical nursing pathway model can deepen the recognition of nursing work among patients with HICH. The main reason is that a multidisciplinary management plan based on the clinical nursing pathway model can improve the functional rehabilitation effect of HICH patients, reduce the occurrence of complications, ensure good disease outcomes, and alleviate the problems caused by the disease in patients’ physical and mental health and daily life, resulting in higher satisfaction.^[20]^

Limitations: This was a single-center study with few observational indicators and no long-term follow-up. Further research is needed to verify the long-term effects in patients.

## 5. Conclusion

In summary, our findings indicate that adopting a multidisciplinary management approach based on clinical nursing pathways to intervene in patients with HICH can reduce stress response levels, reduce the risk of complications, and facilitate the recovery of neurological function and activities of daily living with high patient satisfaction. Because of the limitations of the study, the findings should be interpreted with caution.

## Author contributions

**Conceptualization:** Lan Zhang.

**Data curation:** Tingting Shen.

**Formal analysis:** Yan Zhou.

**Investigation:** Xing Xie.

**Software:** Xing Xie, Jing Wang.

**Writing – original draft:** Lan Zhang.

**Writing – review & editing:** Lan Zhang, Haixiao Gao.
